# The Occurrence of Intersex in Different Populations of the Marine Amphipod *Echinogammarus marinus* in North-West Brittany – A Longterm-Study

**DOI:** 10.3389/fendo.2021.816418

**Published:** 2021-12-24

**Authors:** Matthias Oetken, Marissa Adler, Katharina Alt, Jean Bachmann, Andrea Dombrowski, Franziska Duhme, Anna-Louise Gabriel, Judith Grünewald, Jonas Jourdan, Maren Lück, Carola Mensch, Dominik Rösch, Anna Ruthemann, Susanne Terres, Maja Lorina Völker, Ferdinand Wilhelm, Jörg Oehlmann

**Affiliations:** ^1^ Department Aquatic Ecotoxicology, Goethe University, Frankfurt am Main, Germany; ^2^ Pharmaceuticals, German Environment Agency (UBA), Dessau, Germany; ^3^ Department System Ecotoxicology, Helmholtz Centre for Environmental Research (UFZ), Leipzig, Germany; ^4^ Karlsruhe Institute of Technology (KIT), Karlsruhe, Germany; ^5^ German Federal Institute of Hydrology (BfG), Koblenz, Germany

**Keywords:** crustacea, effect monitoring, intersexuality, sediment, endocrine disruption, range boundary

## Abstract

In the past two decades, an increasing body of studies has been published on the intersex phenomenon in separate-sexed crustaceans from marine and freshwater ecosystems. Various causes are being considered that could have an influence on the occurrence of intersex. Besides genetic factors, environmental conditions such as photoperiodicity, temperature, salinity and parasitism, but also environmental pollution with endocrine disrupting chemicals (EDCs) are discussed. As part of a long-term monitoring (2012 – 2020) in north-west Brittany, we recorded the occurrence of intersex in the marine amphipod *Echinogammarus marinus*. We quantified the intersex incidence at marine and estuarine sites and analyzed the incidence in relation to the endocrine potential of the sediments. Intersex occurred with mean frequencies between 0.87% and 12%. It was striking that the incidence of intersex increased with increasing distance from the sea. Since the highest incidence was observed at the range boundary of this stenohaline species, we assume that intersex is triggered by endocrine potential and increasing stress due to increasing freshwater content − and thus an interplay of different environmental factors.

## Introduction

The term intersexuality was used for the first time at the beginning of the 20^th^ century and describes in the animal kingdom the atypical expression of both female and male secondary sexual characteristics of an individual of a gonochoristic (separate-sexed) species (1). Within aquatic invertebrates, about 340 taxa are known to exhibit the intersex phenomenon, most of which belong to the phyla Mollusca and Arthropoda (2). Among these, aquatic gastropods represent a clade particularly frequently affected by intersex, with about 256 species. Intersex also occurs relatively frequently in the Crustacea. For example, Ford & Fernandez (3) list at least 20 amphipod species from freshwater, estuarine, and marine ecosystems that may exhibit intersex. To date, no specific trigger for intersex has been identified (4). Several causes are considered for the occurrence of intersex. In addition to genetic factors (5), environmental conditions such as photoperiodicity (6), temperature, salinity, and parasitism (7) have also been considered. Environmental pollutants, especially endocrine disruptors (EDC), are discussed as another trigger (8, 9). Martins et al. (10) consider a form of environmental sex determination (ESD) triggered by endocrine disruptors as responsible factor for the occurrence of the intersex phenomenon.

In the present long-term study, we focus on the amphipod species *Echinogammarus marinus*, which is commonly found in marine and estuarine ecosystems across Europe. Intersex individuals of this species develop oostegites as well as one or two penile papillae. Externally, a distinction can be made between intersex males and intersex females, with intersex females developing one or two penile papillae in addition to their female phenotype and intersex males developing brood plates in addition to the male phenotype (4). Here, we refrained from further differentiation between intersex males and intersex females on the basis of phenotypic characteristics, because in some cases a clear identification of the intersex type is difficult. Some individuals exhibited both well-developed oostegites and clearly visible penile papillae. Bulnheim (7) categorized five different types of intersexuality for the euryhaline species *Gammarus duebeni*, classified on the basis of primary and secondary sexual characteristics. However, the author pointed out difficulties of classification for the transitional types. Also other authors like Dunn et al. (11) found a range of intersex expressions, which could not be limited to two intersexuality types.

We focused on north-west Brittany between the city of Roscoff in the East and the southern bay of Brest and monitored over nine consecutive years the occurrence of intersex and non-intersex animals, to compare it with the intersex frequency of *E. marinus* populations in other regions, for example on the east coast of Scotland (12). To interpret the findings, ecotoxicological tests were performed to estimate the impact of environmental pollutants, in particular EDCs, in order to find a link between the occurrence of intersex and chemical exposure. Because *E. marinus* is essentially a marine species that has limited tolerance to lower salinity (13), we suppose that populations occurring in estuarine or even riverine areas at the margin of their salinity range are subject to increased osmotic stress. This stress may be strengthened, by environmental pollutants deposited especially in estuarine or riverine areas, and these stressed populations become more susceptible to parasites. Overall, we hypothesize that populations living at the range boundary are less resilient to the induction of intersex compared to purely marine populations.

## Materials and Methods

### Study Sites and Sampling

**Figure 1:**
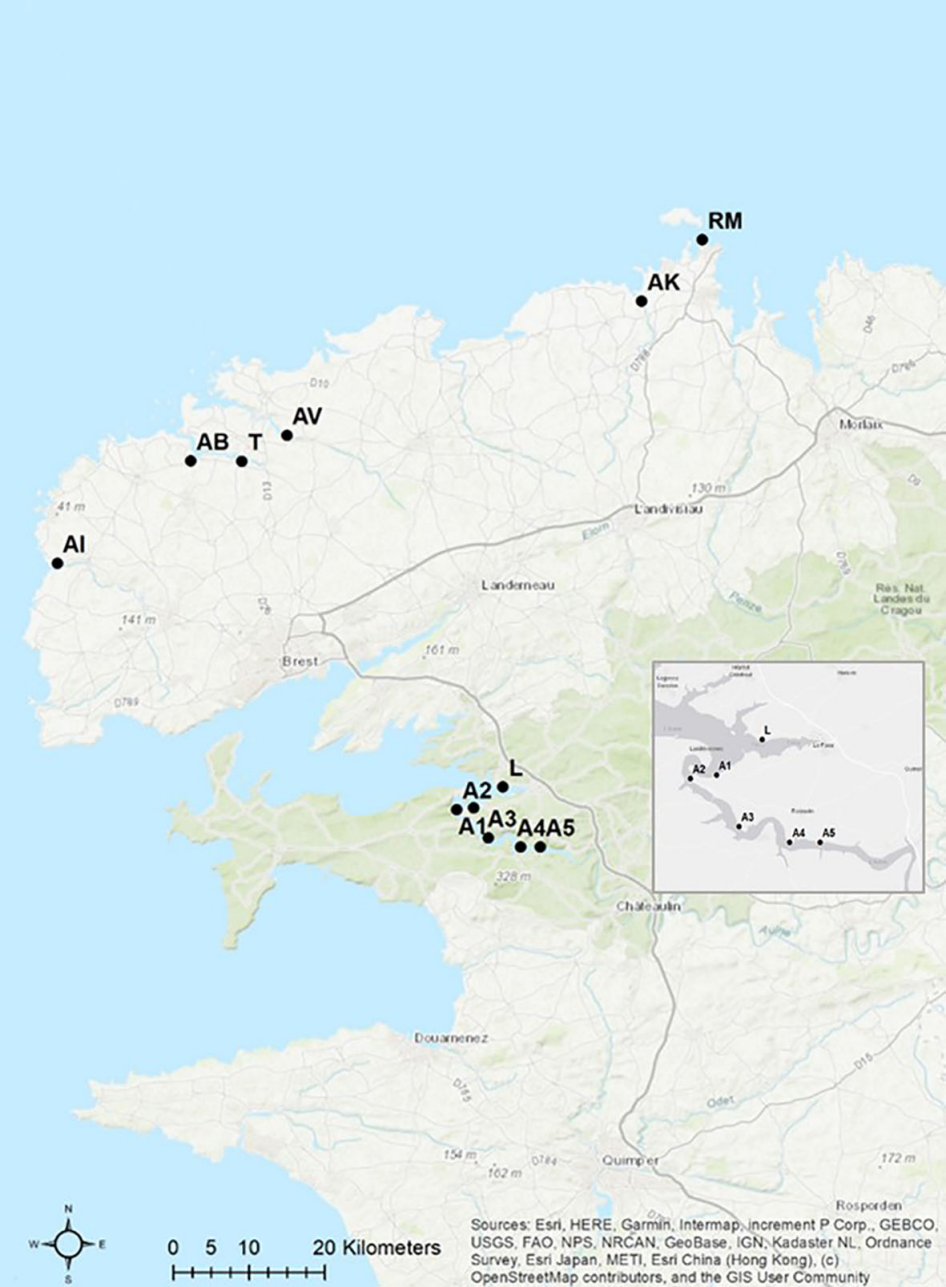
Long-term monitoring 2012-2020. Sampling sites in north-west Brittany (map modified from ArcGis, **Table 1** for abbreviations).

Long-term monitoring was carried out once a year in March from 2012 to 2020 at 12 sampling sites in north-west Brittany (**Table 1**). The sampling sites were located between the Bay of Brest and the city of Roscoff. Sampling sites include a variety of habitat types, ranging from sites directly at the sea, to estuarine regions and inland rivers (**Figure 1**). Due to the fact that *E. marinus* is a relatively stenohaline marine species (13), site Roscoff (RM) was chosen as a reference because it is directly adjacent to the open sea. In addition, this sampling site is relatively uncontaminated with environmental pollutants (14). At each sampling site, between 56 and 192 gammarids per year (a total of 11,355 amphipods during the long-term monitoring) were collected by hand capture during low tide and then transferred individually into 2 mL Eppendorf tubes containing 80% ethanol.

**Table 1:** Sampling sites in north-west Brittany, indicating site abbreviations, distance from the sea, coordinates and number of samples taken.

Sampling site	Site abbreviation	Distance from the sea [km]	Coordinates (UTM-WGS84)	Sampling quantity [years]
**Roscoff Mauer**	RM	0	E 427261; N 5397642	8
Aber Kerbrat	AK	0	E 421915; N 5392360	8
Aber Ildut	AI	1.2	E 370397; N 5370361	9
Aber Benoît	AB	7.9	E 382278; N 5379081	7
Tréglonou	T	9.9	E 386752; N 5378965	8
Aber Vrac’h	AV	11.2	E 390720; N 5381126	7
Lanvoy	L	30.8	E 409118; N 5350000	7
Térénez	A1	30.5	E 406523; N 5348202	9
A2	A2	33.2	E 405037; N 5348079	2
Rosnoen	A3	37.1	E 407754; N 5345547	7
A4	A4	43.2	E 410604; N 5344689	2
A5	A5	45.2	E 412330; N 5344670	2

### Biomonitoring With *Echinogammarus marinus*

*Echinogammarus marinus* belongs to the family Gammaridae and the order Amphipoda. The species prefers to reside among algae and small stones in the sediment and tolerates different salinities only to a very limited extent (13). As detritus feeders on the one hand and fish prey on the other, amphipods play an important role in the food web (15). *E. marinus* is a separate-sexed species that exhibits sexual dimorphism. Females have an average smaller body length than males as well as oostegites (brood plates) between their second and fifth coxal plates of the peraeopods with setae forming the marsupium (brood chamber). Males have penis papillae ventrally between the last pair of peraeopods. Once *E. marinus* was uniquely identified (16), intersex incidence was determined. There are different approaches to characterise intersex in amphipods: by behaviour, by external morphology, by endocrine physiology and by gonadal differentiation (11). Due to time limitations, we only used external morphological characters for intersex detection. Furthermore, we recorded the brood size of normal females and intersex individuals and the body length [basis of first antenna to end of telson (8)]. To account for possible body size effects of breeding individuals, we determined the fecundity index (quotient of offspring and body length) (17).

### Sediment Analyses

Sediment samples were collected from 2015-2020 at each site using a shovel. The topmost one cm of sediment was collected into 3 L freezer bags, deep frozen in order to kill the in fauna and freeze-dried (Christ, Osterode). Freeze-drying was applied instead of drying the samples in a furnace to avoiding the loss of thermally instable contaminants. To analyse the sediment samples, methanol extracts of the sediments were prepared to assess baseline toxicity (Microtox assay), mutagenicity (Ames assay) and endocrine potential (yeast reporter gene assays) of the sediments. For alcoholic extraction, 10 g of homogeneously mixed and freeze-dried sediment from each sampling site was shaken with 50 mL methanol (HPLC grade) for one hour at 210 rpm (GFL, Schwerte, Germany). Subsequently, the sediment-methanol suspensions were transferred to an ultrasonic bath for 10 min and then centrifuged at 4,400 rpm (Eppendorf, Hamburg, Germany) for 5 min. The methanolic supernatant was filtered through 0.1 µm glass microfiber filters and then concentrated in a rotary evaporator (Heidolph, Schwabach) at 56°C and in a second step under a gentle stream of nitrogen. Finally, the extracts were dissolved in 500 µL DMSO, concentrated again with nitrogen and then deep frozen.

### *In-Vitro* Assays

To detect non-specific and specific toxicity of sediment samples, we used the Microtox assay, the Ames fluctuation test and recombinant yeast screens. The baseline toxicity was assessed using the Microtox assay with the bioluminescent bacterium *Aliivibrio fischeri* according to Harth et al. (18). Control samples (negative, procedural blanks), a reference compound (3,5- dichlorophenol) and sediment extracts were tested in triplicates. Results are expressed as EC_50_ values corresponding to the amount of sediment sample (sediment equivalent – EQ) which induced a 50% inhibition of luminescence. An EC_50_-threshold of 100 mg sediment-EQ was defined for samples that reached less than 20% inhibition of luminescence which means they have no toxic potential. This threshold value represents the lowest EC_50_ of a non-toxic sample. The measured activities in the assays are reported as mean ± standard error of the mean (SEM) from three independent experiments.

The mutagenicity in the extracted sediment samples was assessed using the Ames fluctuation test with the *Salmonella typhimurium* strains YG1041 and YG1042 with and without metabolic activation (S9 mix), respectively according to Giebner et al. (19). The test strains used in the Ames test are unable to synthesize the amino acid histidine due to mutations in the histidine operon. Through a reverse mutation, bacteria of such a strain can regain the ability of the original wild type. Since the probability of such a reverse mutation increases after exposure to mutagens, the number of so-called revertants can be used as a measure of the mutagenicity of the environmental sample. The strains YG1041 and YG1042 are characterized by an additional plasmid (pYG233) with a nitroreductase and acetyltransferase gene in comparison to the standard strains TA 98 (frame shift mutation) and TA 100 (base pair mutation). Due to the overexpression of the enzymes O-acetyltransferase and nitroreductase, these strains are more sensitive to mutagens from the group of nitrated aromatic hydrocarbons (nitrosamines, aromatic amines, nitroarenes). *Salmonella* strains were exposed for 100 min to extracted samples in triplicate in 24-well microplates. 2-Nitrofluorene and 2-aminoanthracene served as positive controls for the strains YG1041 and YG1042, respectively. After incubation, the exposure media were diluted by reversion indicator medium with bromocresol purple as pH indicator. Cultures were transferred to 384-well plates (48 replicates per sample) and incubated for 72 h at 37°C. Mutagenicity is expressed as number of revertant wells of the sample in percent.

Recombinant yeast-based reporter gene (*lacZ* encoding for β-galactosidase) assays were conducted to assess the endocrine activity according Stalter et al. (20). In this study the yeast estrogen (YES), androgen (YAS), anti-estrogen (YAES) and anti-androgen (YAAS) assays were used. Assays were performed in 96-well plates with eight technical replicates for each sample, including controls, procedural blanks and reference compounds. To detect antagonistic activities, 17β-estradiol (YAES) and testosterone (YAAS) were added as agonists to the medium. We measured each sample three times (in case a sample showed no activity after the second measurement, the third measurement was omitted). Results are provided as equivalent concentrations (ng/g or mg/g) of the following reference agonists or antagonists: Estradiol equivalents (E-EQs) were calculated for estrogenic activity, testosterone equivalents (T-EQs) for androgenicity, 4-hydroxytamoxifen equivalents (OHT-EQs) for anti-estrogenicity, and flutamide equivalents (F-EQs) for anti-androgenicity. The limit of quantification (LOQ) amounted to 549 ng EEQ/g for the YES, 52.6 ng T-EQ/g for the YAS, 1710 mg OHT-EQ/g for the YAES and 310 mg Flu-EQ/g for the YAAS.

The number of years sediment samples were analyzed between 2015 and 2020 varied from 1 to 6 (n(RM)=6, n(AK)=6, n(AI)=5, n(AB)=6, n(T)=6, n(AV)=6, n(L)=6, n(A1)=6, n(A2)=2, n(A3)=5, n(A4)=2, n(A5)=1).

### Statistical Analyses

Statistical analyses were performed using GraphPad Prism version 5.03 for Windows (GraphPad Software, San Diego, California, USA). For significant differences, we tested data for normal distribution (D’Agostino and Pearson omnibus normality test) and equality of variances (Bartlett’s test). If these conditions were met, the data were tested for significant differences compared to the reference RM using a one-way ANOVA followed by a Dunnett’s Multiple Comparison test. Otherwise, a non-parametric test (Kruskal-Wallis Test, followed by a Dunn’s Multiple Comparison-Test) was used (* = p < 0.05). Pearson product-moment correlation was applied to determine the strength of the linear relationship between two variables.

## Results

### Biomonitoring With *Echinogammarus marinus*

#### Intersex Incidence

**Figure 2:**
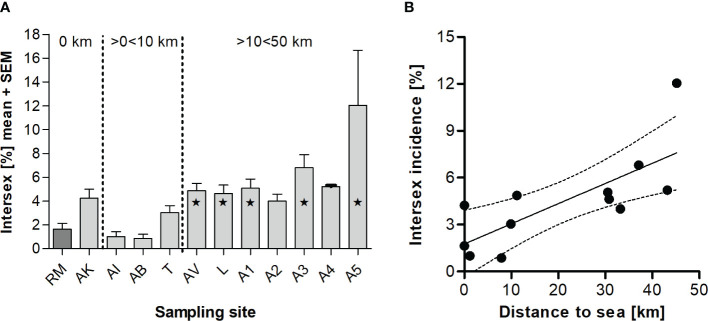
Mean intersex incidence (+ SEM) in *Echinogammarus marinus* at different sampling sites in NW-Brittany (France) during long-term-monitoring from 2012 to 2020 **(A)**. Total numbers of intersex specimens: n(RM)=19, n(AI)=14, n(AK)=52, n(AB)=9, n(T)=36, n(AV)=56, n(L)=51, n(A1)=64, n(A2)=13, n(A3)=71, n(A4)=16, n(A5)=24. Statistically significant differences to reference RM are marked by asterisks (* = p < 0.05, one-way ANOVA with Dunnett’s Multiple Comparison-Test). Linear regression between the mean intersex relative incidence (2012–2020) in *Echinogammarus marinus* and the distance to the sea (p < 0.01, Pearson r = 0.749) **(B)**.

We found low intersex incidences of 0.868% (AB) - 4.24% (AK, 52 out of 1,233 animals) at sampling sites directly at the sea as well as in estuarine regions (i.e., ≤ 10 km away from the sea), which did not differ significantly from RM (**Figure 2A**). Interestingly, with increasing distance to the coast (≥ 10 km), we determined higher intersex incidences (AV with 4.87% (56 out of 1,150 animals), L with 4.64% (51 out of 1,102 animals), A1 with 5.07% (64 out of 1,264 animals), A3 with 6.81% (71 out of 1,042 animals) and A5 with 12.0% (24 out of 201 animals)). As shown in **Figure 2B**, we observed a significant correlation between the intersex incidence at the different sampling sites and their distance from the coast (Pearson r = 0.749, p < 0.01). The farther the sampling sites were from the coast, the higher the intersex incidence of the *E. marinus* populations.

#### Reproduction

**Figure 3:**
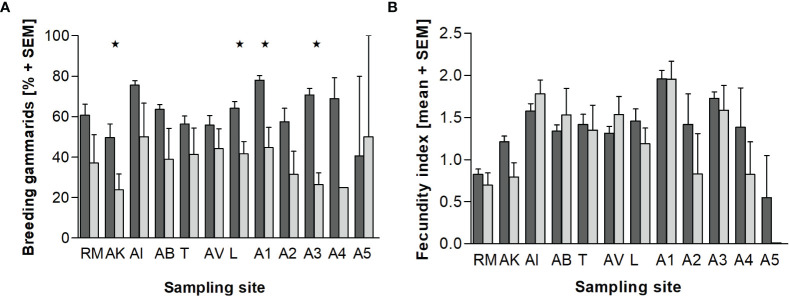
Long-term monitoring in NW-Brittany (2012-2020). Percentage of breeding animals **(A)** and fecundity index **(B)** of non-intersex (dark gray) and intersex (light gray) *Echinogammarus marinus*. Statistically significant differences between normal and intersex specimens are marked by asterisks (* = p < 0.05; *t* test). For abbreviations of the sampling sites see **Table 1**.

Intersex and non-intersex individuals of *E. marinus* that carry offspring are displayed for all sampling sites in **Figure 3A**. At the reference site RM 60.7% of non-intersex females, and 37.0% of intersex individuals carried offspring. Overall, intersexes tended to have a lower proportion of breeding animals compared to non-intersex individuals, but the differences were statistically significant only at sample sites AK, L, A1, and A3. Offspring numbers, expressed as a fecundity index (number of eggs or embryos divided by the body length of the gammarid) are shown in **Figure 3B**. We observed mean fecundity indices ranging from 0.55 (A5) to 1.96 (A1) in the non-intersex and from 0.69 (RM) to 1.96 (A1) in the intersex individuals, with no statistically significant differences between the non-intersex and the intersex individuals.

#### *In-Vitro* Assays

**Figure 4:**
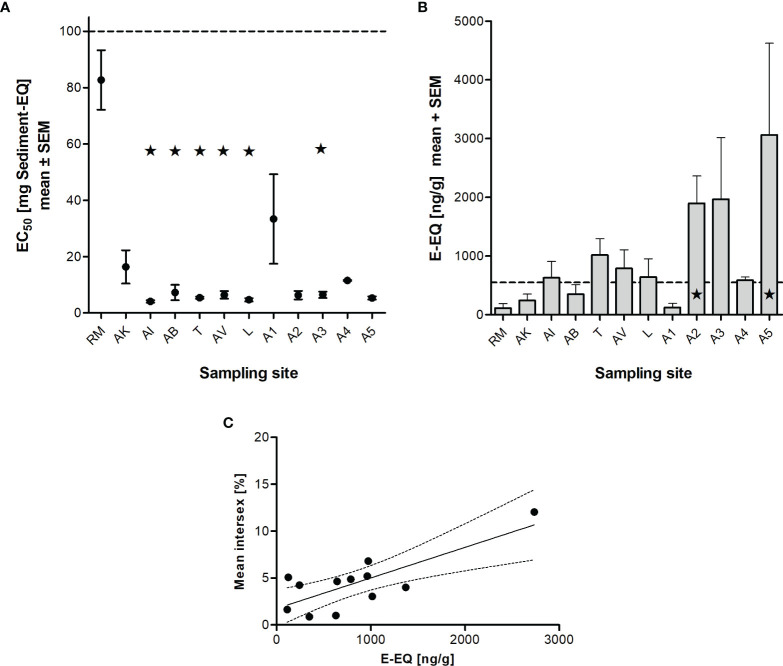
Unspecific and specific toxicity of sediment samples collected in NW-Brittany, France (2012-2020). Microtox assay, expressed as EC_50_ values (mean ± SEM) **(A)**, estrogenic potential, expressed as estradiol equivalents (EEQ) from sediment extracts (limit of quantification (LOQ) 549 ng/g EEQ) **(B)**, and relationship between the mean intersex incidence and the estrogenic potential (YES) of sediments (p < 0.01, Pearson r = 0.779) **(C)**. Statistically significant differences to reference RM are marked by asterisks (* = p < 0.05; Kruskal-Wallis Test with Dunn’s Multiple Comparison-Test). For abbreviations of the sampling sites see **Table 1**.

The sediment at the reference site RM showed by far the lowest toxicity in the Microtox assay with an EC_50_ value of 82.7 mg sediment equivalent (**Figure 4A**). The sample site AK, however, indicated a higher non-specific toxicity with an EC_50_ of 16.4 mg sediment equivalent. Sample sites AI, AB, T, AV, and L were significantly more toxic compared to our reference RM (Kruskal-Wallis Test with Dunn’s Multiple Comparison-Test). At these sites, between 4.08 mg of sediment (AI) and 7.26 mg of sediment (AB) were sufficient to reduce bacterial luminescence by 50%. The remaining sites also showed a remarkable higher baseline toxicity compared to RM.

**Figure 4B** shows the estrogenic activity of sediment extracts from north-west Brittany as estradiol equivalents in nanograms per gram of dry weight sediment. The estrogenic potential of the sediments at both the reference RM (113 ng/g EEQ) and the sites AK, AB, and A1 (243 ng/g EEQ, 347 ng/g EEQ and 124 ng/g EEQ) was below the limit of quantification (549 ng/g EEQ). The estrogenicity in the sediments of the sites AI, T, AV, L and A4 was just above the LOQ. At site A3 the estrogenic activity was clearly increased with 1.97 µg/g E-EQ. Finally, the sediments from sampling sites A2 and A5 indicated significantly increased estrogenic activity with 1.89 µg/g E-EQ and 3.06 µg/g E-EQ, respectively, compared to the reference site RM. **Table 2** shows further the results of the *in vitro* assays investigating the mutagenic potential (Ames fluctuation assay with strain YG1042 with and without S9-mix) as well as the androgenic (YAS), anti-androgenic (YAAS) and anti-estrogenic potential (YAES) of the sediments. Here, we found no trend with increasing distance from the sea. Overall, all sediments can be considered non-mutagenic (both with the strain YG1041 and YG 1042 with and without S9-mix), as the proportion of revertant colonies remains below 20%. While no androgenic activity was detected in the sediments at sites RM, T, AV, and A2, it was highest at site A5 with 39.8 T-EQ ng/g, but still below the limit of quantification of 52.6 T-EQ ng/g. YAAS activity is also below the LOQ (310 Flu-EQ mg/g) and no sediment showed significantly increased activity compared to reference site RM. The same is true for YAES, with only A3 showing significantly increased anti-estrogenic potential (Kruskal-Wallis test with Dunn’s Multiple Comparison test) compared to RM.

**Table 2:** Specific toxicity (mean ± SEM) of sediment samples collected in NW-Brittany, France (2012-2020) (LOQs: YAS: 52.6 ng T-EQ/g, YAAS: 310 mg Flu-EQ/g, YAES: 1710 mg OHT-EQ/g, *=p<0.05).

Site	Ames (YG 1041) % revertant colonies		Ames (YG 1042) % revertant colonies		YAS T-EQ [ng/g]	YAAS Flu-EQ [mg/g]	YAES OHT-EQ [mg/g]
	-S9	+S9	-S9	+S9			
**RM**	5.20 ± 1.76	7.64± 2.56	9.72± 3.35	4.17 ± 1.61	0	0	20.3 ± 210
**AK**	5.20 ± 1.99	6.59 ± 1.74	5.21 ± 1.92	4.17 ± 2.35	5.50 ± 9.19	0	0
**AI**	7.08 ± 1.69	16.3 ± 4.76	6.67 ± 1.79	10.8 ± 3.32	19.1 ± 21.5	128 ± 123	73.5 ± 185
**AB**	6.25 ± 3.53	6.94 ± 2.06	7.99 ± 1.97	9.72 ± 3.71	8.59 ± 11.4	0	139 ± 179
**T**	8.68 ± 2.18	10.2 ± 2.41	8.33 ± 2.58	14.2 ± 3.29	0	15.7 ± 57.8	534 ± 294
**AV**	6.94 ± 3.68	7.98 ± 3.97	5.55 ± 1.58	7.64 ± 3.07	0	0	1985 ± 175
**L**	14.2 ± 2.89	13.2 ± 5.65	8.68 ± 1.47	6.59 ± 1.47	6.80 ± 11.8	138 ± 87.5	178 ± 338
**A1**	4.17 ± 0.932	9.72 ± 1.39	8.68 ± 2.11	10.1 ± 2.48	30.3 ± 22.6	34.5 ± 65.6	81.6 ± 287
**A2**	6.25 ± 0	9.38 ± 1.05	5.21 ± 3.13	4.17 ± 2.09	0	35.1 ± 123	679 ± 693
**A3**	8.75 ± 2.22	10.4 ± 2.19	7.08 ± 4.83	12.9 ± 2.13	27.2 ± 39.5	72.3 ± 55.5	2028 ± 548*
**A4**	6.25 ± 0	6.25 ± 4.17	7.29 ± 7.29	12.5 ± 2.08	28.0 ± 11.1	63.9 ± 140	657 ± 365
**A5**	2.08 ± 0	10.4 ± 0	2.08 ± 0	10.4 ± 0	39.8 ± 132	0	236 ± 313

## Discussion

### Intersex Incidence in North-West Brittany and Possible Causes

While in other parts of Europe, intersex incidence in *E. marinus* ranged from 10% (Iceland) to 18.6% (Scotland) (21), our analysis of the 2012-2020 data series revealed an intersex incidence between 0.87 (AB) and 12.0% (A5) for north-west Brittany. Conceivably, salinity could influence the intersex incidence of this intrinsically stenohaline species. The farther a sample site is from the coast, the lower the average salinity of the water will be. However, salinity was not determined at any of the sample sites because accurate measurement is difficult due to the high variability of salinity at a single site over the tidal cycle (22). The fluctuating salinity in the estuaries is caused by the alternation of high and low tide, so that it is difficult to find a suitable time to measure a reliable value. However, due to the geographical location of the sites, it can be assumed that the water directly on the coast has a higher salinity than the water in the Bay of Brest or along a river gradient such as the L’Aulne River (A2-A5). Since site RM is located directly at the seacoast, it can be assumed that the water has the average salinity of Atlantic water. Site A3, on the other hand, is located in the estuary of the L’Aulne, about 37 km from the open sea. Therefore, the water at site A3 is likely to have a comparatively lower average salinity. Based on this, it can be assumed that the population at A3 is exposed to lower salinity resulting in a higher stress level compared to the population at site RM. Terres (23) found lethal effects on *E. marinus* caused by a reduced salinity (1.04%) in a chronic laboratory experiment. The control group was kept under Atlantic conditions (3.5%) while several test groups were exposed to lower salinities. *E. marinus* showed significantly increased mortality as water salinity decreased.

In addition to lower salinity, parasites at sampling sites further from the sea could act as additional stressors for gammarids. For example, microsporidia are suspected to cause intersexuality (2). According to Dunn and Hatcher (24), microsporidia are transmitted from females to their offspring. However, high salinity levels appear to prevent transmission of microsporidia during follicular maturation which reduced infections of the amphipod *G. duebeni* at high salinity habitats. According to Bulnheim (25), microsporidia appear to be sensitive to high salinity such that they can no longer exert an influence on the sexual development of infected gammarids. Consequently, gammarids living at sampling sites near the sea and at higher salinities may be less likely to carry parasites and are therefore less likely to exhibit intersexual characteristics. This means in reverse that populations remote from the sea are more likely to show higher intersex incidences due to increased parasite infestation. To support this statement, we characterized 5 males and 5 females from each sampling site, as well as all available intersexes using PCR analysis (data not shown). Universal microsporidian primers were used for analysis and tested for the genus *Dictyocoela* and for unicellular parasites of the *Paramyxea* group. The results demonstrated that the prevalence for both groups of parasites was significantly higher in the intersex individuals compared to the non-intersex individuals. Males had a prevalence for *Paramyxea* of 7% (*Dictyocoela* was not detected), females of 20% (*Dictyocoela* 4%), and intersex animals of 44% (*Dictyocoela* 33%). The outcome is fairly consistent with studies previously conducted in the UK on *E. marinus* (26). There, 86% of intersex males were infected with *Paramyxea*, compared to 3% of non-intersex males.

Another trigger that is related to the prevalence of intersex is environmental pollution, which could act as a further stressor, particularly at sampling sites farther away from the sea. In estuaries especially sediment-bound pollutants concentrate that are discharged to rivers diffusely (primarily from agriculture) or through point sources such as wastewater treatment plants (27). To determine non-specific toxicity of the sediments, we used the Microtox assay and found that our reference site RM had the lowest toxicity and all other sites (with the exception of AK and A1) indicated high toxicity. If we consider the specific toxicity of the sediments, an estrogenic potential could be detected in the estuaries and especially along the L’Aulne (A1 to A5). In contrast, neither a genotoxic, nor androgenic, antiandrogenic or even antiestrogenic potential of the sediments was found. To identify potential cause of this finding, Lück (14) analyzed the sediments for polycyclic aromatic hydrocarbon (PAH) and heavy metal contamination and found at the reference site a total content of all 16 EPA PAHs of 0.8 mg/kg dw, while the total concentration was about twice as high at A3 with 1.55 mg/kg dw. Concentrations of heavy metals were also significantly higher at A3 (for example, As was increased 8-fold, Pb 46-fold, and Cd 75-fold compared to RM). This is in agreement with findings of Ford et al. (28), who collected more than 5,300 *E. marinus* in two consecutive years and found intersex incidence twice as high at sites polluted by runoff from the petrochemical industry and at a site located near a ship scrapping facility and a paper mill, compared to an unaffected reference site (12% *vs*. 6%). Enhanced levels of PCBs and heavy metals had been detected there. In a previous study, up to 15% of *E. marinus* exhibited intersex at the polluted sites (29). Ford (30) reviewed the findings of other authors linking pollution to intersex incidence. Within pollutants, special attention is paid to endocrine disruptors. Endocrine disruptors can cause adverse effects on development and reproduction in aquatic organisms. In particular, synthetic estrogens, such as insecticides, plasticizers and polychlorinated biphenyls, are known to act like or enhance the effects of natural estrogens (27–29). In our study, we were able to show *via* the biological effect monitoring that the estrogenic potential of sediments determined in the YES significantly correlated with the intersex incidence in *E. marinus* during 2015-2020 (see **Figure 4C**). This is initially a statistical correlation that must certainly be interpreted with due caution. Presumably, no direct correlation can be established between the intersex incidence and the estrogenic potential of the sediments, since crustaceans do not possess an estrogen receptor (31). Furthermore, neither Daphnia (32) nor the amphipod *E. marinus* (33) have been found to have CYP19 (aromatase) that is critical for aromatization of androgens and thus for biosynthesis of estrogens. However, it may be possible that estrogenic compounds co-occur with other chemicals that may be partly responsible for the development of intersex in *E. marinus*. However, our knowledge so far does not seem to be sufficient to explain the exact mechanisms. Besides the pollutant load and parasite infestation, it is likely that intersex incidences in *E. marinus* are influenced by other interacting environmental conditions. For example, Bulnheim (25), Maranhão et al. (34), and Guler et al. (6) showed that parameters such as photoperiod, temperature, and season also influence the sex differentiation in *E. marinus*. Thus, it is difficult to relate the occurrence of intersex to a single environmental factor, as it is more likely an interaction of multiple factors.

### Intersex and Possible Consequences for Affected Populations

We demonstrated that a lower proportion of intersex individuals are reproductively active compared to normal females (this difference was significant for four of twelve sites). Regarding fecundity, no significant differences between intersex and non-intersex animals were found. In other studies, lower sperm density of *E. marinus* was observed in intersex males (35), while intersex females lost on average about 10% of the brood compared to normal females (12) (possibly due to the deformed oostegites). To illustrate the magnitude of these findings, Ford et al. (36) developed an individual-based population model for *E. marinus*. According to this model, a population at a sampling site died out after 2.3 years if more than 10% of the females exhibit the intersex phenomenon. However, it is important to keep in mind that the affected populations are not isolated, as continuous migration of individuals along the coastline and within estuaries is likely to mitigate losses from high intersex incidences (37). It was found that offspring of intersex and normally sexed individuals are not inferior in fitness to offspring of separately sexed parental pairs (11). According to Maranhão and Marques (38), *E. marinus* has a multivoltine life cycle meaning the species reproduces 2-4 times per year. Assuming an average brood size of about 30 juveniles (38), Guler (6) calculated that a single female has up to 120 offspring per year. Through this high reproductive rate, the r-strategist *E. marinus* compensates for the loss of part of its brood. In this context, a study by Ford and Glazier (39) is also interesting. The authors reported on well-documented populations of *Gammarus minus* in a habitat in Virginia (USA) for over 30 years showing up to 100% intersex. However, despite this unusual and extremely high intersex incidence, reproduction does not appear to be significantly affected and populations persist (39) (40),. The authors conclude that intersex individuals may not have lower reproductive fitness than normal individuals. Similar to *G. minus*, we also assume for the species *E. marinus* that intersex not necessarily jeopardizes reproductive fitness and thus ultimately does not pose a risk for the continuance of an affected population.

## Conclusion

Overall, we provide further evidence that the intersex phenomenon is widespread in *E. marinus* which highlights the value of the species in intersex research. We refrain from identifying unique causal factors for this and assume that the stenohaline *E. marinus* is exposed to an increasing number of different stressors toward the range boundary. Osmotic stress, increasing susceptibility to parasites, and higher endocrine potential are likely to be major factors that − individually or in combination – resulted in an increased incidence of intersex individuals. To identify the importance of these individual stressors, future studies should cover even larger geographic areas so that individual factors (e.g., endocrine potential, osmotic stress) gain local importance and collinearity of environmental variables is reduced. Alternatively, standardized breeding can help by varying environmental factors under laboratory conditions and documenting the eventual emergence of intersex individuals.

## Data Availability Statement

The raw data supporting the conclusions of this article will be made available by the authors, without undue reservation.

## Author Contributions

MO and JO conceived and designed the study. Moreover both drafted and revised the manuscript. JJ also revised the manuscript. AD performed sediment analyses. MA, KA, JB, FD, A-LG, JG, ML, CM, DR, AR, ST, MV, and FW helped with the sampling campaigns, analyzed and interpreted the data. CM collected and cleared the data. All authors contributed to the article and approved the submitted version.

## Funding

This study was funded by the state of Hesse, Germany.

## Conflict of Interest

The authors declare that the research was conducted in the absence of any commercial or financial relationships that could be construed as a potential conflict of interest.

## Publisher’s Note

All claims expressed in this article are solely those of the authors and do not necessarily represent those of their affiliated organizations, or those of the publisher, the editors and the reviewers. Any product that may be evaluated in this article, or claim that may be made by its manufacturer, is not guaranteed or endorsed by the publisher.
